# Dynamic Contrast-Enhanced CT in Patients with Pancreatic Cancer

**DOI:** 10.3390/diagnostics6030034

**Published:** 2016-09-06

**Authors:** Rie Ø. Eriksen, Louise S. Strauch, Michael Sandgaard, Thomas S. Kristensen, Michael B. Nielsen, Carsten A. Lauridsen

**Affiliations:** 1 Department of Diagnostic Radiology, Rigshospitalet, Copenhagen University Hospital, DK-2100 Copenhagen, Denmark; louisesoeborg9@gmail.com (L.S.S.); michael@sandgaard.org (M.S.); tskaarup@yahoo.com (T.S.K.); mbn@dadlnet.dk (M.B.N.); Cala@phmetropol.dk (C.A.L.); 2 Department of Technology, Faculty of Health and Technology, Metropolitan University College, DK-2100 Copenhagen, Denmark

**Keywords:** DCE-CT, pancreatic cancer, diagnostics, treatment response, scan techniques

## Abstract

The aim of this systematic review is to provide an overview of the use of Dynamic Contrast-enhanced Computed Tomography (DCE-CT) in patients with pancreatic cancer. This study was composed according to the PRISMA guidelines 2009. The literature search was conducted in PubMed, Cochrane Library, EMBASE, and Web of Science databases to identify all relevant publications. The QUADAS-2 tool was implemented to assess the risk of bias and applicability concerns of each included study. The initial literature search yielded 483 publications. Thirteen articles were included. Articles were categorized into three groups: nine articles concerning primary diagnosis or staging, one article about tumor response to treatment, and three articles regarding scan techniques. In exocrine pancreatic tumors, measurements of blood flow in eight studies and blood volume in seven studies were significantly lower in tumor tissue, compared with measurements in pancreatic tissue outside of tumor, or normal pancreatic tissue in control groups of healthy volunteers. The studies were heterogeneous in the number of patients enrolled and scan protocols. Perfusion parameters measured and analyzed by DCE-CT might be useful in the investigation of characteristic vascular patterns of exocrine pancreatic tumors. Further clinical studies are desired for investigating the potential of DCE-CT in pancreatic tumors.

## 1. Introduction

Pancreatic cancer is the fourth-leading cause of cancer deaths for both males and females according to estimates for 2016 in the United States [1]. The two most common types of pancreatic cancer arise from either exocrine (95%) or endocrine (5%) cells and can be differentiated by their distinct appearances in vascular patterns [2,3]. Pancreatic ductal adenocarcinoma arises from exocrine glands and is generally characterized as hypovascular [3]. It is by far the most common type [4,5] as it accounts for about 80% of all pancreatic carcinomas [1]. The second most common pancreatic cancer is the neuroendocrine tumor which accounts for about 5% of all pancreatic carcinomas [1]. This type of carcinoma arises from the endocrine glands [4,5,6] and is frequently characterized as hypervascular [3,7]. The prognosis is remarkably lower for exocrine tumors than neuroendocrine tumors with five-year survival rates of 5% and 53%, respectively [1]. Early medical imaging is essential in the investigation of suspected pancreatic cancer in order to reach diagnosis and determine resectability [3,6]. In patients with unresectable tumors, medical imaging plays a key role in the evaluation of treatment response [6]. Multiple imaging techniques as CT, Magnetic Resonance Imaging (MRI), Positron Emission Tomography CT (PET/CT) [3,6,8], transcutaneous ultrasound (US), Endoscopic Ultrasound (EUS) [3,6], and Endoscopic Retrograde Cholangiopancreatography (ERCP) [3] can be used for diagnosis and staging and for evaluation of tumor response to treatment.

According to The European Society of Medical Oncology, a radiological study of a suspected pancreatic carcinoma should include a contrast-enhanced CT scan in both arterial and portal venous phases to assess vascular involvement and metastatic disease [4].

However, it can be difficult to detect pancreatic adenocarcinomas as they may appear isoattenuating to the surrounding parenchyma in the selected contrast phases. Conventional contrast-enhanced CT scans present information about vascular patterns of the tumor in two phases, yet the technique is unable to provide quantification of temporal dynamic changes in perfusion parameters in tumor tissue, as is possible with DCE-CT [9].

DCE-CT provides a noninvasive assessment of perfusion parameters in the scanned volume of tissue [10]. DCE-CT has shown to be a useful biomarker in oncology imaging regarding distinction of diseases and evaluation of response to treatment [10,11]. Analysis of tumor vascularity based on DCE-CT is feasible when characterizing hemodynamic abnormalities and hence distinguishes malignant and benign tumors [12]. Measurements of perfusion parameters are achieved through analysis of temporal changes in attenuation in blood vessels and tissues, caused by intravenous injected iodinated contrast media. Special software and mathematical algorithms are applied when post-processing the data [13,14]. Vascular patterns in tumors can be evaluated and analyzed quantitatively by measurements of perfusion values within a region of interest (ROI) placed in a pancreatic artery and in tumor tissue, or qualitatively by a visualization of perfusion in the respective tissues in a color map [12,15].

The aim of this systematic review is to provide an overview of the use of DCE-CT in patients with pancreatic cancer.

## 2. Materials and Methods

The eligibility criteria and analysis in this review were performed according to the PRISMA guidelines 2009 (Preferred Reporting Items for Systematic Reviews and Meta-Analyses) [16].

The literature search was conducted in PubMed, Cochrane Library, EMBASE, and Web of Science databases to identify publications on DCE-CT in patients with pancreatic cancer. Selection criteria for the included articles were publications written in English and published within the last 10 years (2006–2015). The last search was completed on the 20 April 2016. In the mentioned databases, it was appropriate to apply search terms tailored to the capabilities of each database. As an example, MeSH terms in PubMed were used to specify the search of studies. It was also required to use free text words to include studies, which were not yet assigned to MeSH terms. The following search string was applied: Tomography, X-ray computed[MeSH Terms] OR CT OR “Computed Tomography” AND Perfusion imaging[MeSH Terms] OR DCE-CT OR “Dynamic contrast enhanced” OR Dynamic OR Perfusion OR Functional AND Pancreatic Neoplasms[Mesh] OR Abdominal Neoplasms[MeSH Terms] OR Carcinoma, Neuroendocrine[Mesh] OR “Pancreatic neoplasms” OR “Pancreatic cancer” OR “Neuroendocrine Neoplasms” OR “Neuroendocrine Cancer”.

Screening of studies was performed by two authors (R. Eriksen and C. Lauridsen) who reviewed all titles and abstracts of titles with relevance, from the initial search of the four databases. The same two authors read and selected studies for inclusion. In cases of disagreement, consensus was attained through discussion. Articles on clinical studies concerning DCE-CT in patients with pancreatic cancer were included. Reference lists of all included studies were searched manually for additional literature. 

For all included studies, we extracted and summarized relevant data under the following headings: publication year; study design; number of patients; diagnosis; scan parameters-scanner slice, the amount of contrast, kV and mAs; kinetic model; aim; gold standard; result; conclusion. If required, we also recorded type of treatment and the time at which DCE-CT scans were performed.

To assess the risk of bias and applicability of each included study, the Quality Assessment of Diagnostic Accuracy Studies (QUADAS-2) tool was used. The risk of bias and applicability concerns are defined as the risk to deviate from the QUADAS-2 guidelines described in four domains; patient selection, index test, reference standard, flow, and timing. Both the assessment of risk of bias and concerns about applicability were classified high, low, or unclear [17]. Consensus about the assessment was reached between the same two authors who selected studies for inclusion.

## 3. Results

### 3.1. Study Selection and Division

**Figure 1:**
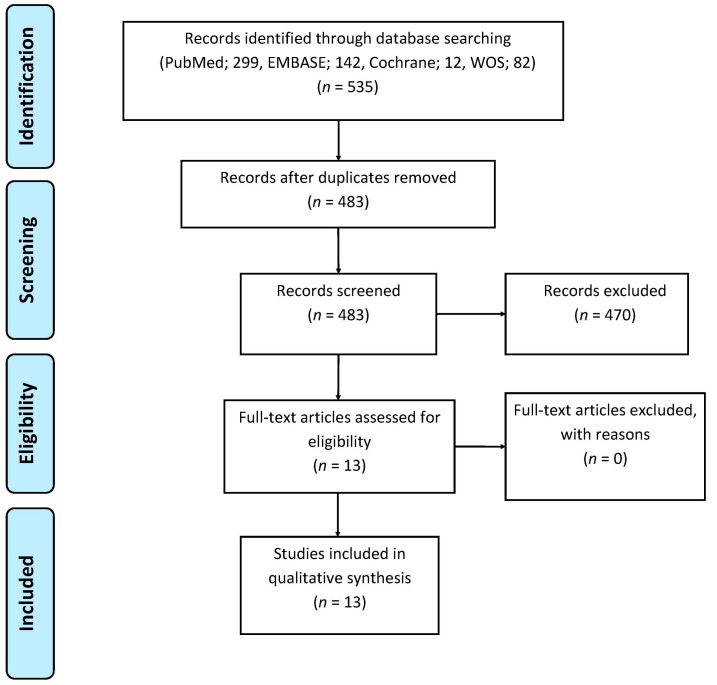
Flowchart of the literature search and study selection.

A PRISMA flowchart of the literature search and final selection of articles is depicted in Figure 1. The initial search yielded 483 publications after duplicates were removed. Four-hundred-and-forty-one articles were excluded on the basis of the title and 29 articles were excluded on the basis of the abstract. All of the remaining 13 articles were included and analyzed after full text reading. Articles were categorized into three groups shown in Appendix A: nine articles concerning primary diagnosis or staging of pancreatic cancer (Table A1), one article about pancreatic tumor response to treatment and three articles concerning scan techniques in pancreatic tumors (Table A2). All 13 articles used a prospective research design.

### 3.2. Studies Concerning Primary Diagnosis and Staging

This group included nine studies (Table A1). Histology was the gold standard for all of the nine studies. In one study, microvessel density (MVD) was used as an additional gold standard [18].

Exocrine pancreatic adenocarcinomas were included in eight of the nine studies [19,20,21,22,23,24,25,26]. In two of the nine studies by D’Assignies et al. [18] and Delrue et al. [20] the patients enrolled had endocrine—or neuroendocrine pancreatic tumors as the primary diagnosis. Delrue et al. included and examined patients with both adenocarcinoma—and neuroendocrine tumors [20].

Blood flow was measured in seven of the eight studies concerning DCE-CT scans in patients with exocrine tumors [19,20,22,23,24,25,26]. In six of the seven studies, the blood flow was significantly lower in tumor tissue compared with pancreatic tissue outside of tumor or normal pancreatic tissue in a control group of healthy volunteers. One study observed higher blood flow in peripheral tumor tissue which showed significant correlation with shorter survival [25].

Blood volume was measured in six of the eight studies concerning DCE-CT scans of patients with exocrine tumors [19,20,21,23,24,26]. In five of the six studies, the blood volume was significantly lower in tumor tissue compared with pancreatic tissue outside of tumor or normal pancreatic tissue in a control group of healthy volunteers [19,20,23,24,26]. One study compared the blood volume between tumors classified as high grade neoplasms and low grade neoplasms, where the blood volume was significantly lower in high grade neoplasms compared with low grade neoplasms [21].

Permeability was measured in five of the eight studies [19,20,23,24,26]. In three of the five studies, permeability was significantly lower in tumor tissue compared with pancreatic tissue outside of tumor or normal pancreatic tissue in a control group of healthy volunteers [19,23,26].

Endocrine tumors were included in two studies. One study showed significantly higher blood flow and blood volume in tumor tissue compared with normal pancreatic tissue in a control group of healthy volunteers [20]. In the second study, blood flow had a significant correlation with microvessel density and the blood flow tended to be higher in tumor tissue compared with pancreatic tissue outside of tumor [18].

Permeability was measured in both studies in tumor tissue and in pancreatic tissue outside of tumor or normal pancreatic tissue in a control group of healthy volunteers, but in none of the studies was a significant difference detected.

### 3.3. Study Concerning Tumor Response to Treatment

In the only study concerning tumor response to treatment patients were diagnosed with exocrine pancreatic tumors and they were treated with concurrent chemotherapy and radiation therapy (Table A2). The gold standard was the WHO classification on responders and non-responders. DCE-CT was used as a baseline scan to measure permeability. First and second follow-up were performed using a two-phase spiral CT to evaluate treatment response. Permeability measured at the baseline DCE-CT was significantly higher in responders than in non-responders [27].

### 3.4. Studies Concerning Scan Techniques

This group included three studies (Table A2). Histology was the gold standard for all of the three studies.

Klauss et al. assessed the feasibility of dual-source CT with measurements at 80 kV, 140 kV, and weighted average of 120 kV, in pancreatic adenocarcinomas. Blood flow and blood volume were measured with three values of kV. Permeability was significantly lower in tumor tissue compared with normal pancreatic tissue in a control group of healthy volunteers at 80 kV and 140 kV [28]. 

Li et al. attempted to evaluate the viability of low-dose DCE-CT on patients with pancreatic adenocarcinomas, divided into two groups and scanned with different levels of kV and mAs, dependent on their weight. Blood flow and blood volume were significantly lower in tumor tissue compared with pancreatic tissue outside of tumor in both groups. No significant differences in values of blood flow or blood volume were observed between the groups [29].

Tan et al. evaluated the usefulness of low-dose DCE-CT by comparing the use of different separated sequences. This study did not specify the type of pancreatic tumor. The patients were divided into three groups, and the groups were scanned with either all sequences, an odd number of sequences or an even number of sequences. Tissue peak and blood flow were significantly lower in tumor tissue compared with tissue surrounding the tumor in all three groups, which could indicate that the tumor was exocrine. There were no significant differences in tissue peak and blood flow in lesion areas of tumor tissue or lesion surrounding areas of tumor between the groups [30].

### 3.5. Healthy Volunteers Included in the Studies Above

In the 13 studies evaluated, perfusion values were measured in normal pancreatic tissue in healthy volunteers in four studies [19,20,24,30] and in one study perfusion values were measured in normal pancreatic tissue in patients with non-pancreatic diseases [26]. In all of the five studies neither blood flow, neither blood volume nor permeability had significantly different values between the different regions of the normal pancreas.

### 3.6. Risk of Bias and Applicability Concerns

Table 1 shows the results from QUADAS-2 with an assessment of risk of bias and concerns about applicability. All studies were considered to have an overall low risk of bias. Though, almost every study was assessed to have high risk of bias in the index test because of the researcher’s knowledge of the pancreatic diseases of the patients before the DCE-CT examinations.

## 4. Discussion

In this systematic review on DCE-CT of pancreatic cancer, all studies were considered to have low risk of bias. When evaluating all the studies investigating exocrine tumors, measurements of blood flow in eight studies [19,20,22,23,24,26,28,29] and blood volume in seven studies [19,20,23,24,26,28,29], were significantly lower in tumor tissue compared with pancreatic tissue outside of tumor or normal pancreatic tissue in control groups of healthy volunteers (Table A1 and Table A2). Since endocrine tumors were only included in two studies and the studies showed different findings in perfusion measurements, it is not possible to make reliable conclusions from these results [18,20] (Table A1). DCE-CT might be an advantageous imaging technique in the investigation of exocrine pancreatic tumors, as it can differentiate between hypovascular patterns in tumor tissue and normal pancreatic tissue, independent of the heterogeneous scan parameters such as the amount of kV and mAs and kinetic models in the included studies. Similar results found in this review regarding exocrine tumors were reported by Chen et al [31]. DCE-CT was performed on 73 patients diagnosed with renal cell carcinomas which are also hypovascular. Chen et al. found that blood flow, blood volume and permeability were significantly lower in tumor tissue compared with renal cortex outside of tumor. The study also observed significant differences in blood flow, blood volume, and permeability between the three subtypes of renal cell carcinoma (clear cell, papillary, and chromophobe). Hence, all three perfusion parameters positively correlated with microvessel density in all subtypes of renal cell carcinoma [31].

Currently, CT is the most frequently used imaging technique in the diagnostic workup of suspected pancreatic carcinomas [3,6]. In addition, EUS can be used as an invasive imaging approach to diagnose suspected pancreatic cancer [4,6,32]. EUS is advantageous because it is more sensitive in early prediction of pancreatic lesions. In combination with fine-needle aspiration, EUS can provide tissue samples, confirm a suspected malignancy, and be helpful in tumor staging [6,32]. Though, the accuracy and sensitivity of using ultrasound is highly operator-dependent [4,6]. A CT scan is beneficial as it has wide anatomic coverage and one radiological study provides information of both local and distant disease [6]. Further, CT has good spatial and temporal resolution and it can evaluate vascular involvement [6]. Standard CT scan protocols of the pancreas, usually consist of a dual-phase enhanced technique [8]. Images of the arterial phase are initiated about 30 seconds after the injection of a contrast media and the portal venous images are obtained about 60 seconds after the injection. To ensure maximum enhancement of the pancreatic vasculature, selecting a rapid injection of about 3–5 mL per seconds is recommended [8]. The pancreatic arterial phase distinguishes pancreatic adenocarcinomas from pancreatic neuroendocrine tumors [8,33], and the portal venous phase defines pancreatic duct dilatation, peripancreatic tissue involvement, and distant metastasis [5,8]. In the arterial phase, a pancreatic adenocarcinoma is depicted as a homogenous hypoattenuating mass with well-defined margins, and opacification of the nearby arteries provides information on vascular involvement [4,5]. In general, a pancreatic neuroendocrine tumor is depicted with hyperattenuating areas in the arterial phase [5,8]. In contrast, DCE-CT only assesses local diseases because the examination is performed over the area covering the suspected cancer. However, since contrast-enhanced CT is only able to assess vascular conditions and tissue attenuation measured over time, DCE-CT might be applicable for diagnostic use as a noninvasive imaging technique to quantify dynamic changes in perfusion, and to provide measurable quantitative and visualized qualitative results of perfusion parameters [15,34]. DCE-CT provides information about different perfusion parameters, which has shown its usefulness in oncological imaging. The functional imaging technique has abilities in differentiating pathological lesions, staging of primary tumors [11,12,14], and in predicting disease free survival [11]. DCE-CT has also shown potential in predicting response to therapy and for therapeutic assessments in tumors [11,12,13,14]. Angiogenesis has a significant role in tumor growth and in the formation of metastasis. Hence, repressing angiogenesis can be used as an approach to inhibit tumor growth [10,35]. Anti-angiogenic therapies have cytostatic effects, which will affect changes in the tumor vasculature rather than changes in tumor size affected by cytotoxic drugs [11]. Since hemodynamic characteristics can be assessed by DCE-CT, this imaging technique is beneficial for evaluating angiogenic activity in tumors, monitoring anti-angiogenic therapies and thus, in predicting treatment response of anti-angiogenic drugs. [10,11,12]. Opposed the noninvasive assessment performed by DCE-CT, histopathologic techniques of microvessel density indexes, used for evaluating angiogenesis and monitoring tumor response, is invasive. A histopathologic technique requires tissue samples for quantification of angiogenesis, and so it does only explore a part of the tumor, which can cause misinterpretations due to possibly intratumoral heterogeneity [10]. CT scanners are widely accessible and, therefore, it would be possible to add a DCE-CT protocol. It is possible for DCE-CT to analyze tumor vascularity and temporal changes in attenuation in vessels and tissues through rapid series of images during intravenous injection of a contrast media, because the relation between the iodine concentrations of a contrast media is linearly proportional to the attenuation values [10,13]. Compared with a conventional CT protocol, a DCE-CT protocol is more time-consuming and increases the radiation burden [22,36]. Additionally, time is required for the researcher to post-process image data. DCE-CT consists of a first pass, a delayed phase or both [37], depending on the kinetic model and desired perfusion parameters. Further, DCE-CT requires repeated scans over the same volume of tissue before, during and after contrast injection [11]. When investigating a suspected pancreatic carcinoma with DCE-CT it is preferable to perform the examination with a contrast agent of high iodine concentration of about 350–400 mg/L [38] and at a high flow rate of 4–6 mL/s to improve maximum contrast enhancement in tissues, and to ensure good signal-to-noise ratio [11,38]. Though, the appropriate level of flow rate and iodine concentration depends on which kinetic model is available for the DCE-CT [36].

The kinetic models used as quantification methods for measuring perfusion parameters vary in capabilities and constraints [36,39]. Perfusion parameters are quantified through kinetic models and thereby both quantitative analysis of physiological parameters, obtained by calculations of the information given by each pixel, and qualitatively analysis of the perfusion visualized through color maps are provided [12]. In six of the nine studies categorized in the group, concerning primary diagnosis and staging, a Maximum-slope model was used as kinetic model [19,20,21,22,24,25] (Table A1). Discrepancy in the amount of contrast media, kV, mAs, and flow rate between the six studies makes it difficult to assess the Maximum-slope model, based on these results. As the advantages of each kinetic model differ it is important to discern their abilities and limitations. The two compartmental models are different in their assumption of the correlation between vascular spaces. The Maximum-slope model perceives the intravascular space and the extravascular space as one compartment, whereas the Patlak model separates the intravascular space and the extravascular space, which make it possible to estimate permeability [11,36]. The compartmental analyses are sensitive to noise, possibly causing miscalculation of perfusion parameters [36,40], which should be taken into account when choosing the level of kV and mAs, since higher levels of radiation doses are required to prevent the impact of noise [36]. Accordingly, the recommended tube current for the Maximum-slope model is 100–200 mAs [36]. Despite the disparate exposure parameters between the six studies concerning primary diagnosis and staging using Maximum-slope, with ranges from 80–120 kV and 20–150 mAs [19,20,21,22,24,25], none of the studies reported that noise affected the image quality or the diagnostic usefulness of DCE-CT. The Deconvolution method is less affected by noise as it includes a complete time series of images [36,38], allowing lower tube current of 50–100 mAs [36]. Its tolerance for higher noise levels makes Deconvolution suitable for measuring lower perfusion values of about <20 mL/min per 100 mL. Since exocrine tumors in the pancreas are depicted as hypovascular, the Deconvolution method could be considered useful for measurements in exocrine tumors. Although, in this review, only one study concerning primary diagnosis and staging applied Deconvolution for measurements of perfusion parameters in exocrine tumors and therefore it is not possible to compare the usefulness of the kinetic model [26]. A previous retrospective analysis by Kaufmann et al. investigated reproducibility of perfusion parameters in healthy pancreatic tissue by comparing the kinetic models using the Maximum-slope model in combination with the Patlak model and the Deconvolution method. The DCE-CT examinations were performed in 41 patients, with a median time interval of 2 days between the first and second DCE-CT and 82 days between the second and third DCE-CT. Results showed that the Deconvolution method was more reliable because of its acceptable deviations in the results at follow-ups [39]. Though, longer scan time required for Deconvolution to collect complete image data, for calculation of perfusion parameters, increases the risk of image misregistration, because of motion artifacts caused by patient movement during the scan [36].

One of the included studies by Kandel et al. [22] showed that the effective dose delivered to patients with pancreatic cancer was higher during DCE-CT (10.1 mSv), compared with conventional single-phase contrast-enhanced CT (4.6 mSv) using the Maximum-slope model. The radiation burden should be kept as low as possible, but the consideration of long-term radiation-induced cancer should be assessed in relation to the patient’s individual prognosis and the potential important information of dynamic changes in vascularity from the DCE-CT [13]. Thus, the optimal values of tube voltage for reducing the radiation burden is 80–100 kV [11,34,36], and according to Lundsgaard et al. the tube current can be as low as 35 mAs [34] independently of the anatomical region. Values of tube voltage and tube current varied between the nine studies categorized in the group concerning primary diagnosis and staging, from 80 kV to 140 kV and from 20 mAs to 150 mAs (Table A1). Considering that no differences were found between the included studies whether the investigators used high dose or low dose protocols to differentiate pancreatic tumors from tissue outside of tumor or normal pancreatic tissue, it might be preferable to use low dose protocols at any examination with DCE-CT of the pancreas [13,41].

Among the 13 included studies less endocrine tumors than exocrine tumors were identified, which might reflect the incidence rates. Only two studies included patients with endocrine tumors, and therefore knowledge about the ability of DCE-CT in pancreatic endocrine tumors is limited. The number of patients enrolled in the included studies ranged from 24 to 112 and the studies showed to be heterogeneous in their choice of scan parameters; scanner slice, the amount of contrast media, kV, and mAs and kinetic models. The lack of standardized protocols for DCE-CT makes it difficult to compare the capability of various scan parameters in the investigation and assessment of different perfusion parameters. Regarding the assessment of risk of bias using QUADAS-2 all studies except one were assessed to have high risk of bias in the index test, because the patients’ diagnoses were known by the researchers’ prior the examination with DCE-CT, which could possibly cause unreliable results.

## 5. Conclusions

In conclusion, in all studies where measurements of blood flow and blood volume in exocrine tumors were compared with pancreatic tissue outside of tumor, or normal pancreatic tissue in control groups of healthy volunteers, perfusion parameters were significantly lower in tumor tissue. In the quality assessment accomplished with QUADAS-2, all studies were considered to have low risk of bias. The assessment of vascularity measured and analyzed by DCE-CT might be of potential use in the investigation of exocrine pancreatic tumors and in the differentiation between pancreatic tumors and normal pancreatic tissue. Further clinical studies are desired for investigating the potential of DCE-CT in pancreatic tumors.

## Figures and Tables

**Table 1:** Quality Assessment of Diagnostic Accuracy Studies (QUADAS-2).

Study	Risk of Bias				Applicability Concerns		
	Patient Selection	Index Test	Reference Standard	Flow and Timing	Patient Selection	Index Test	Reference Standard
D’Assignies et al. 2008 [18]	☺	☹	☺	☺	☺	☺	☺
Delrue et al. 2011 [19]	☹	☹	☺	☺	☺	☺	☺
Delrue et al. 2011 [20]	☺	☺	☹	?	☹	☺	☺
D’Onofrio et al. 2012 [21]	☺	☹	☺	☺	☺	☺	☺
Kandel et al. 2009 [22]	☺	☹	☺	☺	☺	☺	☺
Klauss et al. 2012 [23]	☺	☹	☺	☺	☺	☺	☺
Lu et al. 2011 [24]	☹	☹	☺	☺	☺	☺	☺
Nishikawa et al. 2014 [25]	☹	☹	☺	☺	☺	☺	☺
Xu et al. 2009 [26]	☹	☹	☺	☺	☺	☺	☺
Park et al. 2009 [27]	☺	☹	☺	☺	☺	☺	☺
Klauss et al. 2012 [28]	☺	☹	☺	☺	☺	☺	☺
Li et al. 2013 [29]	☹	☹	☺	☹	☺	☺	☺
Tan et al. 2015 [30]	☺	☹	☺	☺	☺	☺	☺

Risk of bias and concerns regarding applicability of the included studies: ☺ Low Risk; ☹ High Risk; ? Unclear Risk.

## Abbreviations

The following abbreviations are used in this manuscript:

DCE-CT	Dynamic Contrast-Enhanced Computed Tomography
PRISMA	Preferred Reporting Items for Systematic Reviews and Meta-Analyses
QUADAS	Quality Assessment of Diagnostic Accuracy Studies
MeSH	Medical Subject Headings

## Appendix A

**Table A1:** Overview of included studies: *Primary diagnosis and staging*.

Primary Diagnosis and Staging (All Studies were Prospective)										
Authors, Publication Year	No. of Patients	Diagnosis	Scan Parameters			Kinetic Model	Aim	Gold Standard	Results	Conclusion
			Slice	Contrast	kV and mAs					
D’Assignies et al. 2008 [18]	28	Pancreatic endocrine tumors	64	40 mL	100 kV, 100 mAs	Deconvolution/distributed parameter model	To correlate perfusion measurement with MVD and to determine whether perfusion parameters differ between tumor grades.	Histology, MVD, and WHO 2000 criteria ^†^	*Pancreatic endocrine tumors:* BF tended to be higher in tumors than in pancreatic tissue outside of tumor (*p* < 0.06). *Correlation with MVD:* Tumor BF correlated with MVD (*p* < 0.001). *Correlation with WHO:* Significantly higher BF in WHO 1 tumors, than in WHO 2 and WHO 3 tumors (*p* = 0.02).	DCE-CT is feasible in patients with pancreatic endocrine tumors and allows evaluation of tumor angiogenesis.
Delrue et al. 2011 [19]	40	Pancreatic adenocarcinoma (*n* = 20) Normal pancreas in healthy volunteers (*n* = 20)	128 Dual-source CT	50 mL	100 kV, 145 mAs	Maximum slope (single-compartment)	To assess perfusion characteristics in patients with pancreatic adenocarcinoma and to compare with values in normal healthy pancreatic tissue.	Histology	*Pancreatic adenocarcinoma:* Significantly lower BF, BV, and PS in the tumor center than in tumor rim and in pancreatic tissue outside of tumor (*p* < 0.05). *Healthy volunteers*: No significant differences in BF, BV, or PS in the different regions of the pancreas. *Comparison-Patients with pancreatic adenocarcinoma and healthy volunteers*: Significantly lower BF, BV, and PS in the tumor center compared with normal pancreatic tissue in healthy volunteers (*p* = 0.01).	DCE-CT provides added value when investigating tumor vascularization in pancreatic adenocarcinoma, compared with image assessment based on tissue density measurements (HU), and can lead to more accurate diagnosis.
Delrue et al. 2011 [20]	54	Pancreatic adenocarcinoma (*n* = 19), acute and chronic pancreatitis (*n* = 3 + 6), neuroendocrine tumors (*n* = 2), (pseudo)cystic lesions (*n* = 3), normal pancreas in healthy volunteers (*n* = 21)	128 Dual-source CT	50 mL	100 kV, 145 mAs	Maximum slope (single-compartment)	To evaluate whether perfusion parameters can distinguish general pathologies of the pancreas and possibly aid in early diagnosis.	Histology	*Pancreatic adenocarcinoma*: Significantly lower BF and BV in the center of the tumor than in normal pancreatic tissue in healthy volunteers (BF and BV: *p* < 0.01). *Neuroendocrine tumors:* Significantly higher values for BF and BV in tumor tissue compared with normal pancreatic tissue in healthy volunteers (*p* < 0.01). *Acute and chronic pancreatitis*: Significantly lower BF and BV than in normal pancreatic tissue in healthy volunteers (*p* < 0.01) *Healthy volunteers:* No significant differences were found in BF, BV, and PS between head, body, and tail of the pancreas.	Significant decreases in perfusion values in both adenocarcinomas and acute and chronic pancreatitis, and the opposite applies to values in neuroendocrine tumors, which were significantly increased, compared to the control group of healthy volunteers. Different perfusion values can be used as an additional parameter to differentiate pancreatic pathologies.
D’Onofrio et al. 2012 [21]	32	Pancreatic adenocarcinoma. Pathological analysis; low grade (*n* = 12) and high grade (*n* = 20).	64	50 mL	120 kV, 150 mAs	Maximum slope (single-compartment)	To describe DCE-CT features and to assess whether these features correlate with the tumor grading.	Histology	Significantly lower median values of BV and PEI in high grade neoplasms compared with low grade neoplasms (BV: *p* ≤ 0.004 and PEI: *p* ≤ 0.012).	DCE-CT can predict tumor grade of pancreatic adenocarcinoma.
Kandel et al. 2009 [22]	30	Pancreatic adenocarcinoma	320	60 mL	100 kV, 22.5 mAs	Maximum slope (single-compartment)	To evaluate a whole-organ DCE-CT protocol and to analyze perfusion differences between tumor tissue and normal pancreatic tissue.	Histology	Significantly lower BF in tumor tissue compared with pancreatic tissue outside of tumor (*p* ≤ 0.01).	DCE-CT carries the potential to improve detection of pancreatic cancers due to the perfusion differences.
Klauss et al. 2012 [23]	25	Pancreatic adenocarcinoma	64 Dual-source	80 mL	80 kV and 270 mAs 140 kV and 50 mAs	Patlak model (two-compartment)	To evaluate the feasibility of DCE-CT for assessing differences in perfusion values of tumor tissue and normal pancreatic tissue.	Histology	Significantly lower BF, BV, and PS in tumor tissue than in pancreatic tissue outside of tumor (*p* < 0.0001). Significantly higher BF in the head of the pancreas than in the tail, measured in pancreatic tissue outside of tumor (*p* = 0.007).	DCE-CT using the Patlak analysis is feasible. Even isodense tumors could be delineated in the color-coded parameter maps.
Lu et al. 2011 [24]	112	Pancreatic adenocarcinoma (*n* = 64), mass-forming chronic pancreatitis (*n* = 15). Normal pancreas in healthy volunteers (*n* = 33)	64	50 mL	80 kV, 50 mAs	Maximum slope (single-compartment)	To investigate characteristics of pancreatic cancer, mass-forming chronic pancreatitis, and normal pancreas with DCE-CT.	Histology and AJCC 2002 classification system *	*Corrected p values < 0.016 were considered significant in this study.* *Pancreatic adenocarcinoma:* Significantly lower BF and BV in tumor tissue compared with normal pancreatic tissue in healthy volunteers (*p* < 0.016). Significantly higher PS in pancreatic tissue outside of tumor than in normal pancreas in healthy volunteers (*p* < 0.016). *Mass-forming chronic pancreatitis:* Significantly lower BF and BV in mass-forming chronic pancreatitis than in normal pancreatic tissue in healthy volunteers (*p* < 0.016). *Comparison –Pancreatic adenocarcinoma and mass-forming chronic pancreatitis:* Significantly lower BF, BV, and PS values in pancreatic adenocarcinoma than in mass-forming chronic pancreatitis (*p* < .016). *Healthy volunteers:* No significant difference between the head, body, and tail of the pancreas.	DCE-CT is feasible in providing quantitative hemodynamic information of pancreatic adenocarcinoma and mass-forming chronic pancreatitis.
Nishikawa et al. 2014 [25]	17	Pancreatic adenocarcinoma	64	40 mL	80 kV, 20 mAs	Maximum slope (single-compartment)	To investigate the relationship between patient prognosis and perfusion in tumor tissue and peritumoral tissue.	Histology, TNM * and Japanese classification (prognosis)	*Peritumoral tissue:* Significant correlation between AUC peritumoral tissue (AUC of the Time Density Curve) or BF peritumoral tissue and survival days from the date on which perfusion CT was performed (AUC: *p* = 0.04, BF: 0.0005). Higher AUC peritumoral tissue and BF peritumoral tissue values were associated with shorter survival days. *Tumor tissue:* No significant correlation between BF and AUC in tumor tissue and survival days.	Patient prognosis may be related to perfusion in peritumoral tissue observed with DCE-CT.
Xu et al. 2009 [26]	76	Pancreatic adenocarcinoma (*n* = 40). Normal pancreatic tissue in patients with non-pancreatic disease (*n* = 36)	64	50 mL	120 kV, 150 mA (rotation time: N/A)	Deconvolution method	To explore the perfusion characteristics of pancreatic adenocarcinoma and normal pancreatic tissue in patients with non-pancreatic disease.	Histology	*Pancreatic adenocarcinoma:* Significant difference in BF, BV, and PS between tumor tissue, tumor rim, and peripheral pancreatic tissue in pancreatic adenocarcinoma, with gradually increased values from tumor tissue to tumor rim and peripheral pancreatic tissue (*p* < 0.02). *Normal pancreas with non-pancreatic disease:* No significant difference in BF, BV, or PS, between the head, neck, body, and tail. *Comparison between patients with pancreatic adenocarcinoma and patients with non-pancreatic disease:* Significantly lower values of BF, BV, tumor tissue, and tumor rim compared with normal pancreatic tissue in patients with non-pancreatic disease (*p* < 0.05). Significantly lower PS in tumor tissue compared with normal pancreatic tissue in patients with non-pancreatic disease (*p* < 0.05). Significantly higher PS in tumor rim and in peripheral pancreatic tissue of pancreatic adenocarcinoma compared with normal pancreatic tissue in patients with non-pancreatic disease (*p* < 0.05).	DCE-CT can differentiate pathological changes from normal tissue. Therefore, DCE-CT should be considered a potential modality to increase the accuracy of CT diagnosis for pancreatic adenocarcinoma.

Abbreviations: MVD (Microvessel Density); BF (Blood Flow); BV (Blood Volume); ^†^ WHO (World Health Organization) 2000 criteria-WHO 1: Well-differentiated endocrine tumors of benign behavior. WHO 2: Well-differentiated endocrine tumors of uncertain behavior. WHO 3: well-differentiated endocrine carcinomas. WHO 4: poorly differentiated endocrine carcinomas; PS (Permeability Surface); HU (Hounsfield Units); PEI (Peak Enhancement Intensity); * AJCC (American Joint Committee on Cancer); TNM (Tumor, Nodes, Metastasis); AUC (Area under Curve); N/A (Not Available).

**Table A2:** Overview of included studies: Tumor response to treatment and scan techniques.

**Tumor Response to Treatment (Prospective Study)**												
**Authors, Publication Year**	**No. of Patients**	**Diagnosis**	**Scan Parameters**			**Kinetic Model**	**Aim**	**Treatment**	**DCE-CT Scans**	**Gold Standard**	**Results**	**Conclusion**
			**Slice**	**Contrast**	**kV and mAs**							
Park et al. 2009 [27]	30	Pancreatic adenocarcinoma	64	50 mL	100 kV, 100 mAs	Patlak model (two-compartment)	To determine whether DCE-CT parameters, permeability and BV can be used to predict response to concurrent chemotherapy and radiation therapy (CCRT).	CCRT	Baseline. First follow-up: 4–6 weeks. Second follow-up: 10–12 weeks after first follow-up	WHO *, responders (complete or partial response: ≥50% decrease from baseline) and non-responders (progressive disease ≥25% increase in the size of lesion or the appearance of new lesions + those with no change)	*First follow-up (n = 30):* The baseline permeability value was significantly higher in responders than in non-responders (*p* = 0.001). No significant difference between baseline BV in responders and non-responders. *Second follow-up (n = 18):* The results were similar to the first follow-up. The baseline permeability value was significantly higher in responders than in non-responders (*p* = 0.002)	Tumors with high pretreatment permeability values indicating higher intratumoral flow tended to respond better to the CCRT. DCE-CT may be used to predict the tumor response of CCRT in patients with pancreatic cancer.
**Scan Techniques (All Studies were Prospective)**												
**Authors, Publication Year**	**No. of Patients**	**Diagnosis**	**Scan Parameters**			**Kinetic Model**	**Aim**		**Gold Standard**	**Results**		**Conclusion**
			**Slice**	**Contrast**	**kV and mAs**							
Klauss et al. 2012 [28]	24	Pancreatic adenocarcinoma	64 Dual-energy CT	80 mL	80 kV and 270 mAs 140 kV and 50 mAs	Patlak model (two-compartment)	To evaluate the feasibility of dual-energy DCE-CT for assessing the differences in BF, PS, and BV between pancreatic adenocarcinoma and normal pancreatic tissue.		Histology	BF, BV, and PS was significantly lower in tumor tissue than in pancreatic tissue outside of tumor, for both 80 kV, 140 kV, and weighted average 120 kV (BF, BV, and PS: *p* < 0.0001).		The use of dual-energy DCE-CT improves the accuracy of DCE-CT of the pancreas by fully exploiting the advantages of enhanced iodine contrast at 80 kV in combination with the noise reduction at 140 kV. Using dual-energy perfusion data could improve the delineation of pancreatic carcinomas.
Li et al. 2013 [29]	33	Pancreatic adenocarcinoma (*n* = 33)	N/A Dual-source CT	50 mL	70 kV and 120 mAs (<70 kg) 80 kV and 100 mAs (≥70 kg)	Patlak model (two-compartment)	To investigate the feasibility of low-dose whole pancreas DCE-CT.		Histology	*Pancreatic adenocarcinoma:* Significantly lower BF and BV in pancreatic adenocarcinoma compared to the normal pancreatic tissue (*p* < 0.001). *Comparison between weight-dependent scan protocols:* No significant difference in BF, BV, and PS in the normal pancreatic tissue between patients <70 kg and patients ≥70 kg.		The low-dose whole-organ DCE-CT of the pancreas can effectively reduce the radiation dose.
Tan et al. 2015 [30]	67	Pancreatic carcinoma (*n* = 27). Normal pancreas in healthy volunteers (*n* = 40)	640	40 mL	100 kV, 50 mA (rotation time: N/A)	Maximum slope (single-compartment)	To evaluate the feasibility of low-dose scanning: Compare changes of tissue peak and BF in normal tissue, lesions, and surrounding areas. Compare the use of the whole sequence (group 1), odd number (group 2), and even number (group 3).		Histology	*Pancreatic carcinoma:* Significantly lower tissue peak and BF in lesion areas of pancreatic cancer than in lesion-surrounding areas in group 1, 2, and 3 (*p* ≤ 0.001). *Healthy volunteers:* No significant difference between the three pancreatic regions no matter which sequence were applied. No significant difference between the three regions comparing the groups.		By using the method of low-dose whole pancreas perfusion, scan sequences, and radiation dose are halved, and the diagnosis capacity is not impaired.

* WHO (World Health Organization) BV (Blood Volume); BF (Blood Flow); PS (Permeability Surface); N/A (Not Available); kg (kilogram).
